# Seasonality of acute kidney injury in a tertiary hospital academic center: an observational cohort study

**DOI:** 10.1186/s12940-021-00691-5

**Published:** 2021-01-15

**Authors:** Gianmarco Lombardi, Giovanni Gambaro, Nicoletta Pertica, Alessandro Naticchia, Matteo Bargagli, Pietro Manuel Ferraro

**Affiliations:** 1 U.O.C. Nefrologia, Azienda Ospedaliera Universitaria Integrata di Verona, Verona, Italy; 2 U.O.C. Nefrologia, Dipartimento di Scienze Mediche e Chirurgiche, Fondazione Policlinico Universitario A. Gemelli IRCCS, Rome, Italy; 3 Dipartimento Universitario di Medicina e Chirurgia Traslazionale, Università Cattolica del Sacro Cuore, Rome, Italy

**Keywords:** Acute kidney injury, Epidemiological study, Seasonality, Weather conditions

## Abstract

**Background:**

The aim of our study was to describe seasonal trends of acute kidney injury (AKI) and its relationship with weather conditions in a hospitalized population.

**Methods:**

We retrospectively collected demographic (age, sex), clinical (ICD-9-CM codes of diagnosis discharge) and laboratory data (creatinine values) from the inpatient population admitted to Fondazione Policlinico Universitario A. Gemelli IRCCS between January 2010 and December 2014 with inclusion of all patients ≥18 years with at least two values available for creatinine. The outcome of interest was AKI development, defined according to creatinine kinetics criteria. The exposures of interest were the months and seasons of the year; air temperature and humidity level were also evaluated. Log-binomial regression models adjusted for age, sex, eGFR, comorbidities, Charlson/Deyo index score, year of hospitalization were used to estimate risk ratios (RR) and 95% confidential intervals (CI).

**Results:**

A total of 64,610 patients met the inclusion criteria. AKI occurred in 2864 (4.4%) hospital admissions. After full adjustment, winter period was associated with increased risk of AKI (RR 1.16, 95% CI 1.05, 1.29, *p*=0.003). Lower air temperature and higher humidity level were associated with risk of AKI, however in multivariable-adjusted models only higher humidity level showed a significant and independent association.

**Conclusions:**

AKI is one of the most common complications of hospitalized populations with a defined seasonal pattern and a significant increase in incidence during wintertime; weather conditions, particularly higher humidity level, are independent predictors of AKI and could partially justify the observed seasonal variations.

**Supplementary Information:**

The online version contains supplementary material available at 10.1186/s12940-021-00691-5.

## Background

Acute kidney injury (AKI) is a complex clinical syndrome characterized by an abrupt reduction of renal function [1]. It is a common and severe complication in hospitalized populations. The reported incidence is quite varied especially since it is strongly influenced by definition and study population with an incidence ranging from 7 to 57% [2–6] and with a significant burden on patient morbidity and mortality [7–9]. As reported by clinical and epidemiological studies, several diseases [10–14] show a defined seasonal variation. “Winter peaks” have been observed in cardiovascular [13], respiratory [14] and infectious [10] diseases.

Understanding the impact of seasonal patterns of diseases could have a role in clinical practice and preventive care. However, despite the importance of AKI, to date only a Japanese retrospective study on an inpatient population has investigated seasonal variations of AKI describing a peak of incidence during wintertime [15]. Although the observed pattern may be in part related to seasonality of underlying illnesses associated with kidney injury, as reported in the aforementioned study, a significant relationship between AKI and wintertime persisted even after adjustment for potential confounders (demographic factors, comorbidities and medications). It may be reasonable to speculate that even environmental factors such as weather conditions might partially influence AKI occurrence.

Therefore, the aim of our study was to describe trend and seasonality of AKI in an Italian cohort of hospitalized patients and to investigate the possible relationship between AKI and weather conditions.

## Methods

### Study population

We performed a retrospective observational cohort study on the inpatient population admitted to Fondazione Policlinico Universitario A. Gemelli IRCSS (Rome, Italy), a tertiary level hospital serving more than 1 million people in Rome collecting patients all around the city, between January 1, 2010 and December 31, 2014. We included only adult patients (18 years or older) with at least two measurements available for serum creatinine (Cr) during hospital stay. Patients with End Stage Kidney Disease (ESKD) were excluded. For patients with multiple hospital admissions, only the first one was considered.

### Data collection

All clinical, demographic and laboratory data were extracted from the electronic hospital database. We exported the following variables for each hospital admission: age, sex, ICD-9-CM codes of primary and secondary discharge diagnoses, Cr, date of hospital admission. Using the Italian meteorological historical records [16] we extracted the following weather variables, at city level, during the same period: average monthly air temperature and relative humidity levels.

### Definitions

AKI diagnosis and severity were defined according to creatinine kinetics (CrK) criteria [17] as an absolute increase of Cr concentration during hospitalization. To do this, we calculated the difference between each Cr and the previous measured value during hospitalization. According to CrK criteria, we defined AKI as an absolute increase in Cr of 0.3 mg/dL over 24 h or a 0.5 mg/dL increase over 48 h.

ESKD was identified according to ICD-9-CM codes in primary and secondary discharge diagnoses.

Estimated glomerular filtration rate (eGFR) was evaluated in all patients at hospital admission by the Chronic Kidney Disease Epidemiology Collaboration (CKD-EPI) equation [18].

The seasons were classified according to the month of the year as spring (March, April, May), summer (June, July, August), autumn (September, October, November) and winter (December, January, February).

ICD-9-CM codes in primary and secondary discharge diagnoses were used to identify comorbidities (cardiovascular diseases, endocrine/metabolic diseases, malignancies, genito/urinary diseases, infectious diseases, respiratory diseases). Charlson/Deyo comorbidity index score [19] was calculated for each patient using ICD-9-CM codes in primary and secondary discharge diagnoses.

The outcome of interest was AKI development (reported as categorical variable [yes/no] in the regression models). The exposures of interest were the months/seasons of the year; secondly, we evaluated air temperature and relative humidity level as exposures. Covariates assessed to control for confounding were age, sex, baseline eGFR, comorbidities, Charlson/Deyo index score, year of hospitalization.

### Statistical analysis

Continuous variables were reported as means and standard deviations (SD) or medians and interquartile ranges (IQR) as appropriate. Categorical variables were described using numbers and percentages (%). Student t-test and chi-square (χ^2^) test were used to compare continuous and categorical distributions in the descriptive analysis between two groups; two-way ANOVA was performed for multiple groups comparisons (three or more groups).

Log-binomial regression models adjusted for all covariates were used to estimate risk ratios (RRs) and 95% confidential intervals (CIs) of months/seasons (June and summer were selected as the reference groups because it contained the smallest proportion of patients with AKI among hospitalized patients). Because AKI occurrence vary largely across different age and sex groups, and hence the seasonal pattern of AKI in different groups may vary, stratified analysis by sex (male vs female) and age (more or less 60 years old) were performed.

Multivariable regression models were subsequently fitted to explore the association between weather conditions (analyzed as continuous and categorical variable grouped in quartiles) and AKI. Three models were built: Model 1 represents unadjusted RRs; Model 2 was adjusted for all covariates (age, sex, eGFR, comorbidities, Charlson/Deyo score, year of hospital admission); Model 3 was adjusted for relative humidity and mean temperature in addition to factors included in Model 2. Ultimately, mediation analysis was performed to evaluate the “mediator role” of weather conditions on AKI seasonality and estimate the *average causal mediation effect* (ACME) as measurement of the amount of the indirect (or mediate) effect or as the amount of the reduction of the causal variable effect (direct effect, [season period]) on the outcome of interest. Confidence intervals were calculated with bootstrapping approach and a high number (500) of simulations. We use the linear regression fit with least squares and the binomial regression for the mediator and outcome models, respectively.

Statistical analyses were performed using R version 3.4.4 (Free software Foundation, California).

A *p*-value < 0.05 was considered as statistically significant.

## Results

### Descriptive measurements of study population

**Fig. 1:**
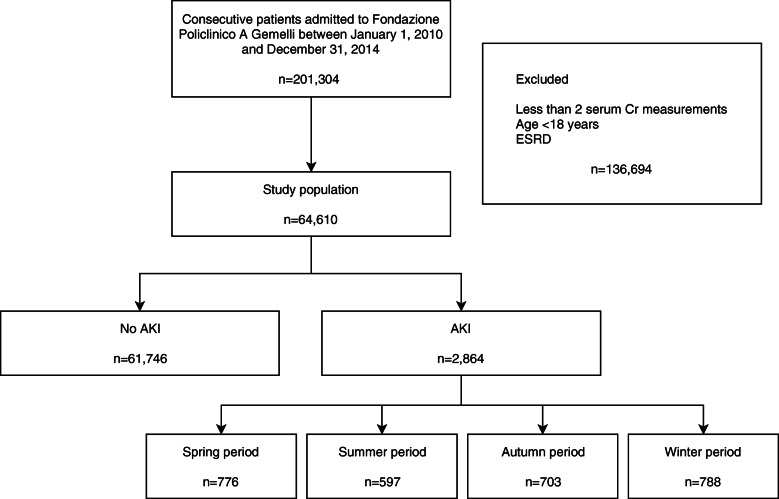
Flowchart of the study

Overall, 64,610 out of 201,304 patients met our inclusion criteria (Fig. 1). AKI occurred in 2864 (4.4%) patients.

Patients with AKI had higher comorbidity index score (0.8 [1.1] vs 0.3 [0.8], *p*< 0.001) with an increase prevalence of cardiovascular diseases (1747 [61.0%] vs 23,208 [37.6%], *p*< 0.001), endocrine/metabolic diseases (702 [24.5%] vs 10,680 [17.3%], *p*< 0.001), genito/urinary disorders (782 [27.3%] vs 6876 [11.1%], *p*< 0.001), infectious diseases (269 [9.4%] vs 3007 [4.9%], *p*< 0.001), respiratory diseases (698 [24.4%] vs 7580 [12.3%], *p*< 0.001). AKI was also more commonly observed in older individuals (70.7 [13.9] years vs 60.2 [18.0] years, *p*< 0.001) and males (1771 [61.8%] vs 28,149 [45.6%], *p*< 0.001) (Table 1).

**Table 1:** Descriptive characteristics of study population stratified by AKI

	No AKI	AKI	*p* value
*n*	61,746	2864	
Age, years, mean (SD)	60.2 (18.0)	70.7 (13.9)	< 0.001
Sex, Males, *n* (%)	28,149 (45.6)	1771 (61.8)	< 0.001
Charlson/Deyo score, *n* (%)			< 0.001
0	48,693 (78.9)	1662 (58.0)	
1	9190 (14.9)	624 (21.8)	
2	2199 (3.6)	319 (11.1)	
> 2	1664 (2.7)	259 (9.0)	
Charlson/Deyo score, mean (SD)	0.3 (0.8)	0.8 (1.1)	< 0.001
Comorbidity, *n* (%)			
Cardiovascular	23,208 (37.6)	1747 (61.0)	< 0.001
Gastrointestinal	9487 (15.4)	410 (14.3)	0.134
Malignancies	19,471 (31.5)	758 (26.5)	< 0.001
Endocrine/Metabolic	10,680 (17.3)	702 (24.5)	< 0.001
Genito/Urinary	6876 (11.1)	782 (27.3)	< 0.001
Infectious	3007 (4.9)	269 (9.4)	< 0.001
Respiratory	7580 (12.3)	698 (24.4)	< 0.001
eGFR, ml/min/1.73m^2^,median (IQR)^*^	82.0 (35.0)	50.0 (43.0)	< 0.001

^*^ eGFR at hospital admission

Overall 17,213 patients were admitted during spring period, 14,674 during summer period, 16,330 in autumn and 16,393 in winter. As reported in Table 2, patients hospitalized during spring and winter period showed a higher comorbidity index score and a higher prevalence in cardiovascular diseases. In patients admitted during winter period we observed an higher prevalence in respiratory diseases. Summertime was instead characterized by a higher prevalence in infectious diseases.

**Table 2:** Descriptive characteristics of study population stratified by season

	Spring	Summer	Autumn	Winter	p value
*n*	17,213	14,674	16,330	16,393	
Age, years, mean (SD)	60.8 (17.8)	60.7 (18.2)	60.1 (18.0)	61.0 (17.9)	< 0.001
Sex, Males, *n* (%)	8005 (46.5)	6831 (46.6)	7475 (45.8)	7609 (46.4)	0.463
Charlson/Deyo score, *n* (%)					< 0.001
0	13,332 (77.5)	11,556 (78.8)	12,870 (78.8)	12,597 (76.8)	
1	2631 (15.3)	2190 (14.9)	2379 (14.6)	2614 (15.9)	
2	701 (4.1)	538 (3.7)	624 (3.8)	655 (4.0)	
> 2	549 (3.2)	390 (2.7)	457 (2.8)	527 (3.2)	
Charlson/Deyo score, mean (SD)	0.4 (0.9)	0.3 (0.8)	0.3 (0.8)	0.4 (0.9)	< 0.001
Comorbidity, *n* (%)					
Cardiovascular	6780 (39.4)	5506 (37.5)	6135 (37.6)	6534 (39.9)	< 0.001
Gastrointestinal	2675 (15.5)	2232 (15.2)	2480 (15.2)	2510 (15.3)	0.8
Malignancies	5365 (31.2)	4536 (30.9)	5160 (31.6)	5168 (31.5)	0.526
Endocrine/Metabolic	3122 (18.1)	2533 (17.3)	2805 (17.2)	2922 (17.8)	0.067
Genito/Urinary	2011 (11.7)	1733 (11.8)	1992 (12.2)	1922 (11.7)	0.453
Infectious	876 (5.1)	816 (5.6)	778 (4.8)	806 (4.9)	0.01
Respiratory	2241 (13.0)	1790 (12.2)	1945 (11.9)	2302 (14.0)	< 0.001
eGFR, ml/min/1.73m^2^, median (IQR)^*^	81.0 (37.0)	79.0 (37.0)	81.0 (36.0)	82. 0 (36.0)	< 0.001

^*^ eGFR at hospital admission

### Association between seasonality and AKI

AKI was more commonly observed during wintertime [788 (4.8%)] compared with other seasons [spring 776 (4.5%), summer 597 (4.1%), autumn 703 (4.3%)] (Table 3). As reported in Table 3, the winter period was significantly associated with higher risk of AKI (RR 1.18, 95% CI 1.07, 1.31, *p*=0.002) (Table 3). This association was confirmed even after correction for potential confounders (RR 1.15, 95% CI 1.04, 1.38, *p*=0.006). No significant relationships were observed after comparing other seasons to each other (Supplementary Table 1). Stratified analysis, by age and sex, demonstrated a significant and independent increase in AKI risk during wintertime only in male subjects older than 60 years (Supplementary Table 2).

**Table 3:** Association between AKI and seasons

	Spring	Summer	Autumn	Winter
*n*	17,213	14,674	16,330	16,393
AKI	776 (4.5)	597 (4.1)	703 (4.3)	788 (4.8)
RR (95% CI)	1.11 (1.00, 1.23) *p*=0.054	1.00 (Reference)	1.06 (0.95, 1.18) *p*=0.300	1.18 (1.07, 1.31) p=0.002
RR (95% CI)^#^	1.09 (0.99, 1.21) *p*=0.100	1.00 (Reference)	1.07 (0.97, 1.19) *p*=0.200	1.16 (1.05, 1.29) *p*=0.003

^#^Adjusted for age, sex, *eGFR* comorbidities, Charlson/Deyo score, year of hospital admission

**Fig. 2:**
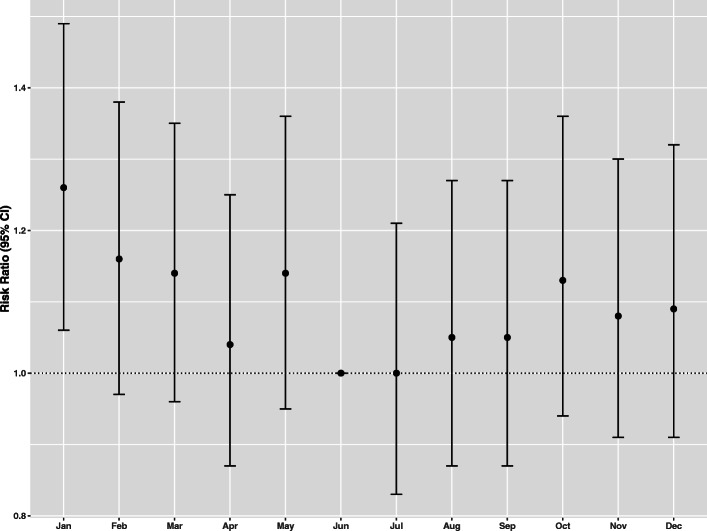
Multivariable-adjusted risk ratios (95% confidence of interval) of AKI associated with month of the year; adjusted for age, sex, comorbidities, eGFR, year of hospital admission, Charlson/Deyo score; June used as reference

The risk of AKI was significantly higher in January (RR 1.28, 95% CI 1.08, 1.57, *p*=0.004) [Fig. 2]) than in the rest of the year.

### AKI and weather conditions

**Fig. 3:**
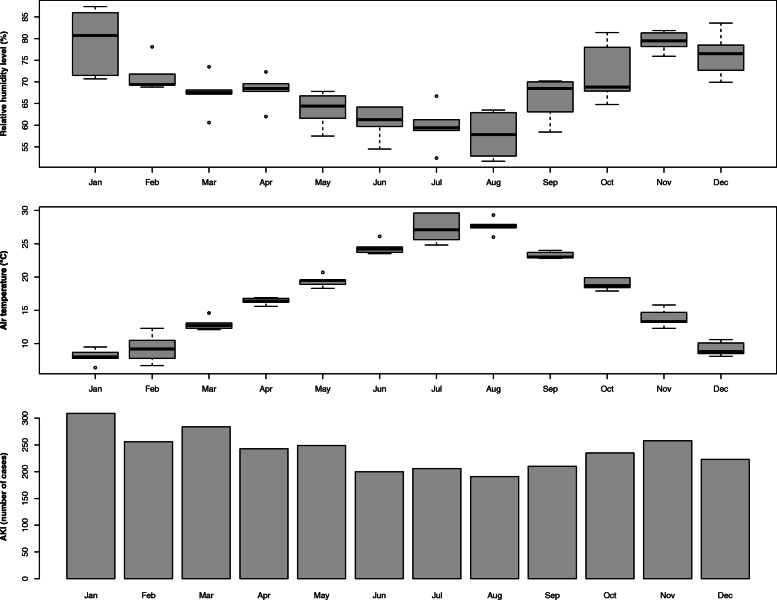
Distribution of AKI cases, relative humidity levels and air temperature over the months

As depicted in Fig. 3 an association between air temperature and relative humidity level can be describe. Progressive increase in the average monthly relative humidity level and a progressive decrease in the average monthly air temperature was associated with increased AKI occurrence (Fig. 3).

Multivariable regression model demonstrate a significant and association between air temperature and relative humidity level with the risk of AKI (RR 1.16, 95% CI 1.05, 1.28, *p*=0.004, in the highest quartile of humidity level compared with the lowest, *p*-value for trend=0.003; RR 1.15, 95% CI 1.04, 1.28, *p*=0.008, in the lowest quartile of air temperature compared with the highest, p-value for trend=0.009; Table 4).

**Table 4:** Association between weather conditions and AKI

	No. of patients	No. of events (%)	Model 1		Model 2		Model 3	
			RR (95% CI)	*p* value for trend	RR (95% CI)	*p* value for trend	RR (95% CI)	*p* value for trend
Humidity level (%)								
- Q1: < 63.0	16,777	656 (4.5)	1.00 (Reference)	< 0.001	1.00 (Reference)	0.003	1.00 (Reference)	0.064
- Q2: 63-68.2	15,643	711 (4.5)	1.16 (1.05, 1.29) *p* = 0.005		1.17 (1.06, 1.30) *p* = 0.002		1.17 (1.05, 1.31) *p* = 0.006	
- Q3: 68.2-72.8	16,497	774 (4.7)	1.20 (1.08, 1.33) *p* < 0.001		1.20 (1.08, 1.32) *p* < 0.001		1.18 (1.03, 1.35) *p* = 0.016	
- Q4: > 72.8	15,693	723 (4.6)	1.18 (1.06, 1.31) *p* = 0.002		1.16 (1.05, 1.28) *p* = 0.004		1.15 (1.01, 1.32) *p* = 0.038	
Air temperature (°C)								
- Q1: < 12.1	16,476	787 (4.8)	1.19 (1.07, 1.32) *p* = 0.002	< 0.001	1.15 (1.04, 1.28) *p* = 0.008	0.009	1.03 (0.89, 1.19) *p* = 0.735	0.675
- Q2: 12.1-16.9	16,603	742 (4.5)	1.11 (1.00, 1.24) *p* = 0.056		1.08 (0.98, 1.20) *p* = 0.136		0.99 (0.87, 1.13) *p* = 0.867	
- Q3: 16.9-23.8	17,424	767 (4.4)	1.09 (0.98, 1.22) *p* = 0.100		1.10 (0.99, 1.22) *p* = 0.081		1.01 (0.89, 1.14) *p* = 0.901	
- Q4: > 23.8	14,107	568 (4.0)	1.00 (Reference)		1.00 (Reference)		1.00 (Reference)	

Model 1: unadjusted

Model 2: Multivariable regression model adjusted for age, sex, eGFR, comorbidities, Charlson/Deyo score, year of hospital admission

Model 3: Fully adjusted model, multivariable regression model adjusted for relative humidity and mean temperature in addition to factors included in Model 2

However, in multivariable model fully adjusted for weather factors (Model 3, Table 4), only humidity level (RR 1.15, 95% CI 1.01, 1.82, *p*=0.038, in the highest quartile of humidity level compared with the lowest) was independently associated with a higher risk of AKI.

In order to evaluate the “complete mediation effect” played by humidity we performed a mediation analysis (after controlling for correlation between humidity level and winter period, β=9.29, *p*< 0.001) that confirmed the significant mediator effect (or indirect effect) of humidity level (ACME average, point estimate 0.0026, 95% CI 0.0005, 0.0100, *p*= 0.028) with an estimate mediated proportion of 0.52 (as ratio between the indirect “mediated” effect and the total effect) on the relationship between AKI and winter period.

## Discussion

Our study describes the relationship between AKI and seasonality and between AKI and weather conditions, air temperature and humidity level.

The incidence of AKI increases during wintertime; weather conditions, such as air temperature and relative humidity level are associated with AKI; humidity level is an independent predictor of AKI occurrence and could partly explain and mediate the observed seasonal variations.

Seasonality patterns in human illnesses are a well-recognized phenomenon since Hippocrates [20]. In particular, variations in the seasonal patterns of cardiovascular diseases, infectious diseases, respiratory diseases have been already described [10–14]. Most studies reported “winter peaks” in cardiovascular [13], respiratory [14] and infectious [10] related hospitalizations and mortality. A complex interaction between individual susceptibility and environmental factors could justify such relationship.

Recently, a Japanese study [15] was the first to describe a seasonality pattern of AKI. The authors, analyzing a historical cohort of hospitalized patients, documented an increase in incidence rates of AKI during winter period. As reported, an increase in cardiovascular and pulmonary diseases during wintertime, might partly explain such association. Although not investigated, authors concluded that weather conditions should also be considered.

In our study AKI occurred in 4.4% (2864/64,610) of hospitalized population. Cardiovascular diseases were the most common disorders associated with kidney injury. As reported in Table 3 and Fig. 2, and similarly to the aforementioned Japanese study, we observed a “winter peak” in kidney injury. Although this relationship could partly be justified by an increase incidence in common diseases generally associated AKI during wintertime (we found an increased prevalence in AKI patients of respiratory and cardiovascular diseases in winter, [Table 2]), an independent association persisted even after adjustment for all covariates.

In order to better understand this independent seasonal variation, we investigated the possible relationship between kidney injury and weather conditions. We found that higher relative humidity level and lower air temperature were associated with risk of AKI development. However, after multivariable adjustement, only humidity level showed and independent relationship with kidney injury. Furthermore a “mediate” role of humidity level on the relationship between winter period and AKI emerges from the analysis. Even if mediation analysis does not imply causal relationships (unless it is based on experimental design) a significant mediation of humidity on AKI seasonality exists. According to our data about the 52% of the winter effect on kidney event is mediated by the humidity level.

It is difficult to explain such relationship. As for cardiovascular diseases [11, 21] from our study it emerges a complex and significant interaction between environmental conditions (temperature and humidity) and kidney diseases. We can speculate that given the strong association between cardiovascular and kidney diseases, these could follow a similar seasonality pattern and a similar response to weather conditions. Air temperature and humidity level are the major determinants of the human core temperature. As already reported, cold exposure provokes physiological changes potentially harmful for the cardiovascular system [21, 22]. Core temperature decline is followed by peripheral vasoconstriction, sympathetic activation which results in an increase in heart rate and blood pressure. In this way it could have adverse and detrimental effects especially in people with underling or sub-clinical kidney suffering. How humidity could affect the kidney is unclear. However, the association high humidity and low temperature was shown to have the greatest impact on cardiovascular mortality in a Chinese study compared to all other possible combination of temperature and humidity levels [21].

Some limitations in our study must be considered. Ours is a monocentric study with a retrospective design; furthermore, we used ICD-9-CM codes for comorbidities assessment and for risk adjustment. On the other hand, we determined the Charlson/Deyo comorbidity index, a validate score based on ICD-9-CM diagnosis codes. Furthermore, we were unable to define the representativeness of our hospital for database limitations.

Ours is the first study describing AKI seasonality in a European cohort and the first one demonstrating an association between weather conditions (humidity levels) and AKI development.

## Conclusions

We conclude that AKI has a seasonal pattern and a significant increase in incidence during wintertime; the seasonality is not explained by common clinical factors. On the other hand, weather conditions, particularly humidity level, are associated with the observed seasonal variations. It remains to be understood if such an association is independent or due to other unknown variables. The climate and its influence on body physiology could represent a new element to be considered and investigated in AKI epidemiology.

## Supplementary Information

**Additional file 1: Table S1**. Association between AKI and seasons. **Supplementary Table 2.** Association between AKI and seasons, stratified analysis by age and sex

## Data Availability

The datasets used and/or analysed during the current study are available from the corresponding author on reasonable request.

## Abbreviations

AKI: Acute kidney injury
Cr: Serum creatinine
ESKD: End Stage Kidney Disease
ICD-9-CM: International Classification of Diseases, Ninth Revision, Clinical Modification
CrK: Creatinine kinetics
eGFR: Estimated glomerular filtration rate
CKD-EPI: Chronic Kidney Disease Epidemiology Collaboration
