# Supplementary material for: Seasonality of acute kidney injury in a tertiary hospital academic center: an observational cohort study
Source: Environ Health. 2021 Jan 15;20:8. doi: 10.1186/s12940-021-00691-5 (PMC7811228; doi:10.1186/s12940-021-00691-5)
Supplement: Supplementary file 1 — Additional file 1: Table S1. Association between AKI and seasons. Supplementary Table 2. Association between AKI and seasons, stratified analysis by age and sex [file 12940_2021_691_MOESM1_ESM.pdf]

Supplementary Table 1. Association between AKI and seasons

	Spring	Summer	Autumn	Winter
RR (95% CI)	1.11 (1.00, 1.23)<br>p=0.054	1.00 (Reference)	1.06 (0.95, 1.18)<br>p=0.300	1.18 (1.07, 1.31)<br>p=0.002
RR (95% CI)[#]	1.00 (Reference)	0.92 (0.83, 1.02)<br>p=0.109	0.98 (0.89, 1.08)<br>p=0.707	1.06 (0.96, 1.16)<br>p=0.230
RR (95% CI)[#]	1.09 (0.98, 1.20)<br>p=0.109	1.00 (Reference)	1.07 (0.96, 1.18)<br>p=0.225	1.15 (1.04, 1.28)<br>p=0.006
RR (95% CI)[#]	1.02 (0.92, 1.12)<br>p=0.707	0.94 (0.84, 1.04)<br>p=0.225	1.00 (Reference)	1.08 (0.98, 1.19)<br>p=0.122
RR (95% CI)[#]	0.94 (0.86, 1.04)<br>p=0.230	0.87 (0.78, 0.96)<br>p=0.006	0.93 (0.84, 1.02)<br>p=0.122	

[#]Adjusted for age, sex, eGFR, comorbidities, Charlson/Deyo score, year of hospital admission

Supplementary Table 2. Association between AKI and seasons, stratified analysis by age and sex

	Summer	Spring				Autumn				Winter			
		≤60 yr		>60 yr		≤60 yr		>60 yr		≤60 yr		>60 yr	
		M	F	M	F	M	F	M	F	M	F	M	F
RR (95% CI)	1.00 (Ref)	1.05(0.78,1.40) p=0.765	1.00(0.66,1.52) p=0.998	1.12(0.96,1.30) p=0.140	1.13(0.94,1.36) p=0.194	1.18(0.89,1.57) p=0.255	1.09(0.73,1.65) p=0.667	1.04(0.89,1.22) p=0.605	1.08(0.90,1.31) p=0.411	1.16(0.87,1.55) p=0.315	0.99(0.65,1.52) p=0.971	1.27(1.10,1.48) p=0.001	1.09(0.90,1.31) p=0.388
RR (95% CI)#	1.00 (Ref)	1.04(0.78,1.39) p=0.790	0.93(0.62,1.41) p=0.738	1.09(0.94,1.26) p=0.272	1.10(0.92,1.32) p=0.283	1.22(0.92,1.61) p=0.170	1.06(0.71,1.59) p=0.784	1.01(0.87,1.18) p=0.866	1.08(0.90,1.30) p=0.419	1.11(0.84,1.47) p=0.476	0.92(0.61,1.41) p=0.708	1.24(1.07,1.43) p=0.004	1.06(0.88,1.27) p=0.547

#Adjusted for eGFR, comorbidities, Charlson/Deyo score, year of hospital admission
